# Sexual and reproductive health issues facing young people with perinatal HIV infection

**DOI:** 10.1097/QAD.0000000000004274

**Published:** 2025-10-30

**Authors:** Caroline Foster

**Affiliations:** Imperial College Healthcare NHS Trust, London, UK.

**Keywords:** HIV-exposed uninfected infants, perinatally acquired HIV, pregnancy, sexual and reproductive health, sexually transmitted infections, youth

## Abstract

Young people with HIV from birth face unique sexual and reproductive health challenges. They are born into a multigeneration family living with HIV and must navigate adolescence with a stigmatizing sexually transmissible infection where personal disclosure has the potential to disclose the status of family members. The article highlights the need for tailored sexual and reproductive health education, emphasizing the importance of addressing HIV stigma, ensuring access to contraception, and providing support for the disclosure process. While rates of sexually transmitted infections are broadly comparable to their peers, higher rates of human papilloma virus infection and persistence highlight the importance of early prevention strategies, including vaccination and screening although gaps exist in the optimal vaccine schedule and age of screening onset. Rates of intimate partner violence and unplanned pregnancy in adolescence are high, with adverse maternal and neonatal outcomes, including higher rates of preterm birth and low birth weight infants when compared to aged-matched peers. Although vertical transmission rates are reassuringly low, maternal health outcomes are of growing concern, driven by prolonged HIV infection with the long-term complications that negatively impact on pregnancy including comorbidities such as hypertension, diabetes, and renal disease. Research gaps persist, particularly regarding fertility, contraception, pregnancy, and the outcomes for their HIV exposed uninfected infants. To thrive, this unique group of young people require inclusive, nonjudgemental, accessible, evidence-based interventions and services to support their sexual and reproductive health, integrated within holistic HIV care, tailored to their needs and voiced by them.

## Introduction

Since 1990, an estimated 11 million children have been born with HIV and with the successful global role out of pediatric antiretroviral therapy (ART) increasing numbers of children are transitioning through adolescence and entering adult care [1]. In high-income countries (HIC) with early access to ART, the oldest survivors are now entering their fifth decade of life [2]. With access to interventions to prevent vertical transmission (PVT) rates of vertical transmission are frequently below 1% for pregnant people with HIV acquired in adult life living in HIC [3,4]. Furthermore, increasing numbers of young adults born with perinatal HIV (PHIV) are entering relationships and having children of their own, the vast majority living free of HIV [5,6]. However, despite effective interventions to PVT, globally an estimated 120 000 [83 000–170 000] children under 15 years continue to acquire HIV annually, the vast majority living in low/middle-income countries (LMIC) [1,4,7]. While global access to ART has markedly improved, early infant diagnosis and timely initiation to ART remains challenging in many settings [4,7]. Without access to ART, half of all children with PHIV will die before their second birthday, although an estimated 10–15% will survive undiagnosed into adolescence although frequently with significant HIV associated morbidity [8,9]. Hence, in all settings, the oldest individuals with PHIV are those who survived the early years of childhood without ART, potentially with favorable host genetics and immune responses but have experienced years of unsuppressed viraemia and frequently older and more toxic ART regimens [10]. In contrast, many of those born in more recent decades started ART in earlier childhood and are more likely reflective of the emerging adolescent perinatal cohorts globally, important in identifying the consequences of lifelong HIV and ART exposure throughout postnatal, childhood, and adolescent development [11].

This editorial examines the emerging literature on sexual and reproductive health (SRH) outcomes for this unique cohort, born into a family living with HIV and the challenges of negotiating adolescence with a potentially sexually transmissible infection for the first and every subsequent sexual encounter and that sharing your status potentially discloses the status of other family members. The impact of biomedical prevention strategies, including vaccination, contraception, screening for sexually transmitted infections (STIs), and for anogenital cancers in the context of PHIV are reviewed. With increasing numbers of young people born with HIV choosing to start families of their own, pregnancy data are emerging, including the early infant outcomes for a unique cohort of “3rd Generation” HIV-exposed uninfected infants born to people who themselves have lived with HIV their entire lives. Finally, the data gaps in sexual, reproductive, and pregnancy health for this cohort are summarized, highlighting future research questions and the importance of the meaningful inclusion of youth in setting the research agenda to support them in fulfilling their potential as adults, partners, and parents.

## Adolescence and the onset of sexual activity

Young people growing up with HIV require early access to tailored sexual health and relationship education prior to the onset of sexual activity. The majority of data suggest adolescents with PHIV have a similar age of coitarche compared to their HIV-negative peers, however the burden of HIV stigma prevents a minority from entering any sexual relationships with follow up now into their late 20 s [12–15]. Qualitative data suggest the onset of sexual activity in young women with PHIV is influenced by the need for love, intimacy and acceptance, and for some, related to meeting survival needs [16]. Data for young men are lacking. The results of the Partner study established that having an undetectable viral load (<200 copies/ml) on suppressive ART for more than 6 months results in a zero risk of sexual transmission of HIV to partners following condomless sex and were published almost a decade ago in 2016 [17]. Despite this, HIV knowledge around undetectable equals untransmittable (U = U) remains low in some adolescent populations, with only 44% of 15–19 year olds with PHIV in South Africa understanding the concept as recently as 2023 [18]. More starkly only 19.5% of Ugandan youth living with HIV (*n* = 276, mean age 18.9, range 15–24 years, 90% PHIV) were considered to have SRH literacy despite engagement in HIV care and regular contact with health professionals [19]. However, in a French cohort (18–25 years PHIV, interviewed 2022) even with a clear understanding of U = U, with 100% of participants aware of the statement, many continue to worry about the potential of HIV transmission despite an undetectable viral load, highlighting that factual knowledge alone is insufficient and the potential of ongoing transmission worries and the impact on sexual satisfaction [13]. In some settings, healthcare professionals remain reluctant to discuss sexuality and sexual health with adolescents and the importance of youth tailored tools delivered by peer educators has been highlighted by the Committee of African Youth Educators [20–23]. Effective interventions to improve adolescents SRH knowledge are emerging and include the utilization of eHealth, including web-based and mobile phone interventions, graphic story lines, using images to supplement text and peer mentors with recent data showing improved sexual health knowledge, understanding of U = U, and partner disclosure [20–23]. Adolescents cite the intergenerational barriers to effective sexual health education at home, within education and within healthcare settings and the need for open communication, stigma reduction around sexuality and the need for support from both adults in their lives and from peer mentors [23]. Qualitative data suggest there may be sex differences in preferences in the way SRH is delivered, with young men preferring older male services providers, whereas young women preferred female providers nearer to their own age [24]. Even within adolescent trials networks, delivery of youth friendly HIV services with integrated sexual healthcare remain challenging, with 69% of sites providing SRH education, 50% contraception, and 38% cervical screening [25]. The access to SRH education from multiple sources, including home, school, youth HIV clinics, community settings, and by eHealth offer diverse spaces to improve SRH education and preparedness for young people in a wide variety of settings [26].

## Onward disclosure and HIV stigma

For those living with PHIV, in sharing their HIV status with others, they risk disclosing the status of their mother and potentially other family members. Rates of disclosure to sexual partners for this population are variable between studies (22–62%) with a potential trend for increasing rates of disclosure over time [27–29]. In a recent study from Ugandan, almost two thirds of 18–25 year olds living with PHIV disclosed to their most recent sexual partner [27]. The potential positive benefits of HIV disclosure include social acceptance, social support, reduction in stigma, and medication support but are balanced by the potential negatives including the fear of rejection, recrimination, and a wider loss of confidentiality [30–34]. HIV-related stigma remains an enormous challenge for young people, with almost a quarter of youth (mean age 16.8 years; 48% having disclosed to most recent partner) in South Africa experiencing enacted HIV stigma within the previous 6 months, associated with nonfamily (partner, teacher, friend) status disclosure [adjusted odds ratio (AOR) = 2.19, 95% confidence interval (95% CI) = 1.60–3.00] [35]. In the same setting, internalized HIV stigma in adolescent boys with PHIV was associated with increased odds of depression [odds ratio (OR) 1.74], alcohol misuse (OR 2.09), and violence victimization (OR 1.44) and decreased odds of medication adherence (OR 0.60) [36]. HIV stigma is a significant barrier to ART adherence, engagement and retention in care, transition to adult services resulting in increased rates of viral nonsuppression and risk of onward transmission to both partners and offspring [36–38]. There is an enormous body of literature and international guidance on the naming of HIV to a child born with PHIV for parents/carers and for healthcare professionals, that contrasts starkly with the lack of guidance around onward disclosure as those living with PHIV enter adulthood. However, young people, their carers, and healthcare professionals all cite the need for evidence-based interventions to support disclosure with encouraging early data suggesting improved wellbeing with structured disclosure interventions for those living with PHIV that include peer workers [29].

## Sexually transmitted infections

High rates of STIs, unplanned pregnancy, and termination have been reported in youth with PHIV, although early data suggest rates are comparable to exposed uninfected peers, with 10–35% reporting having been ever diagnosed with an STI by 25 years of age [14,39–41]. The acquisition of STIs for those living with PHIV has been associated with older age, female sex, substance use, lower CD4^+^ T cell count, and unsuppressed viraemia, highlighting the coexisting vulnerabilities for those who struggle with their management of their physical health and other risk-taking behaviors including unprotected sex in the context of viraemia [40–42]. Furthermore, for youth living with PHIV in Thailand and Vietnam, median age 19 years, serious adverse events including hospitalization and death were associated with the life time acquisition of high-risk human papilloma virus (HR-HPV) serotypes 16 and 18 [43]. Rates of STIs while comparable to general population, are lower in those with PHIV when compared to aged matched populations who have acquired HIV nonperinatally (NPHIV). US cohorts (mean age 20.6 years, 20% PHIV, 28% female) reported an overall STI rates in males of 65.9 vs. 8.5/100 person-years [PY]; females, 54.7 vs. 17.2/100 PY in NPHIV and PHIV, respectively [42]. While *Chlamydia trachomatis* and anogenital HPV are the most commonly reported STIs in those living with PHIV, regional differences do exist, with *Neisseria gonorrhoea* more common than *C. trachomatis* in young women with HIV (76% PHIV) in Eswatini and associated with a new recent sexual partner, sometimes/never condom use, partner aged more than 25 years, and an HIV diagnosis age at least 15 years and highlights the importance of an awareness of local patterns of STIs and integration of sexual health services within community healthcare [44].

## Human papilloma virus; anogenital infection and prevention

Adults with NPHIV have increased rates of HPV infection and HPV-related disease when compared to the general population and data are emerging in PHIV, much from the Treat Asia Study Group [45,46]. In young women with PHIV, HPV incidence (64 vs. 40%) and persistence (27 vs. 10%) are higher than in aged and ethnically matched peers not living with HIV [47,48]. In young men with PHIV, a similar picture is emerging, with incident infection with HR-HPV of 33 vs. 16 per 100 PY in peers not living with HIV, although SRH data remains sparse for young men with PHIV [49,50].

Cervical cancer resulted in over 350 000 deaths in 2022 and is the leading cause of cancer death in women in 21 of 48 countries in sub-Saharan Africa [51,52]. Women with NPHIV are six times more likely to develop cervical cancer, with deaths from cervical cancer disproportionately affecting younger women, with cervical cancer causing an estimated 20% of orphans who have lost their mother to any cancer [53,54]. Young women with PHIV have higher rates of cervical abnormalities (10–33%) occurring at a younger age compared to women with NPHIV [14,55–57]. However, the optimal timing of onset of cervical screening for this specific population is unknown with no recommendations made in global guidelines and needs to be urgently addressed [50,55]. While HPV vaccination is highly effective, whether the reduced HPV vaccine schedules for the general population, with a move to single dose vaccination in early adolescence, will provide adequate sustained protection for those with PHIV is unclear, with a number of ongoing studies addressing this issue [58,59]. In Thai adolescents (9–15 years) on suppressive ART (CD4^+^ cell count >500 cells/μl), a two-dose regimen was as immunogenic as a three-dose schedule [60]. However, waning immunity 6 years post a three-dose HPV quadrivalent vaccination is reported in youth from Zimbabwe, with 17% having HR-HPV serotypes 16/18 with loss of cross protection against HPV45 [61]. The need for, interval, and frequency of HPV vaccination boosting after primary immunization for those living with PHIV remains unknown. The WHO endorsement of single-dose HPV vaccination schedules for nonimmunosuppressed individuals potentially requires HIV youth services to facilitate additional HPV vaccines [62]. Data for men with PHIV and for noncervical HPV-related orogenital disease are sparse, with no guidance on the need for and age of onset of anal screening for this group who are likely at an increased risk of HPV persistence with oncogenic potential beyond the cervix [45,50,63,64].

## Contraception and fertility

Rates of unplanned pregnancy remain high in youth populations including those living with PHIV [65]. A consistent picture emerges in all settings where young people continue to struggle to talk about sexual health and contraception to both parents and providers, and face barriers with access to and experiences of stigma and discrimination within SRH services [65–69]. Rates of any contraception use in recent studies for those living with PHIV vary from 65 to 87%, with some cohorts suggesting rates of double contraception (barrier contraceptive + other) being lower in PHIV than NPHIV [14,27,69–71]. Reasons for not using condoms included difficulty in negotiating use for girls with PHIV, as in the general population, and for those living with PHIV having an undetectable viral load and the knowledge of U = U was viewed as a waiver for condom use for some, with prevention of STI acquisition and pregnancy less of a priority [69,72]. While long-acting reversible contraception is preferred by providers for effective pregnancy prevention, negative beliefs around hormonal contraception remain prevalent within the general population with high rates of discontinuation in young people citing side effects, including irregular bleeding, weight gain, and mood disturbance [69,73]. More reassuringly, the global move away from first-line ART based around efavirenz to dolutegravir has eliminated the potential for drug-drug interactions with hormonal contraceptives, and offers young people on ART, hormonal contraceptive options equal to their peers, and the potential to access local contraceptive services with their friends without the need for disclosure in those who struggle with HIV stigma within healthcare settings [74].

While the focus for young people is primarily on prevention of unwanted pregnancy, much less is known re fertility in this population. Data in adults with NPHIV suggest an earlier onset of menopause and increased rates of secondary amenorrhea for some women and an impact sperm motility and morphology for some men [75–78]. Qualitative studies suggest that most young men and women with PHIV plan future parenthood and are confident in the role of suppressive ART to PVT [79–82]. However, data around fertility for this group are extremely sparse with very small single cohort studies suggesting increased potential for abnormal sperm forms in young men with PHIV, and reduced serum anti-Mullerian hormone levels and increased rates of amenorrhea in young women with PHIV [83,84]. While young women with PHIV tend to have children almost a decade earlier than NPHIV in some settings, questions remain regarding fertility duration for an emerging cohort who have yet to reach the age of menopause and require evidence-based information to inform and optimize their reproductive choices [6]. Whether data can be extrapolated from earlier cohorts with PHIV, more likely to have experienced advanced disease, stunting and delayed onset of puberty to the emerging population of adolescents is unknown and highlights the need for ongoing surveillance of the SRH outcomes for young people PHIV following transition to adult care and the importance of data disaggregated by route of HIV acquisition [85–88].

## Intimate partner violence

In their lifetime, almost one in three women and one in five men report experiencing intimate partner violence (IPV) with the onset frequently occurring during adolescence [89–91]. The physical, mental, societal, and economic costs of IPV are substantial and include physical trauma, STIs, miscarriage and termination, substance use, posttraumatic stress, and major depressive disorders [92,93]. The impact of IPV is not limited to the victim alone; childhood exposure to interparental IPV is associated with an increased risk of adverse physical and neurodevelopmental outcomes and risk of IPV in adult life, highlighting the multigenerational cycle of IPV [94,95]. Men and women living with NPHIV are more likely to report IPV with additional impacts on engagement in care, ART adherence, condomless sex and the potential for onward transmission of HIV [96–98]. Reported rates of IPV in PHIV vary; 30% of Ugandan youth, median age 21.7 years, had ever experienced IPV contrasting with the United States where an alarming 84% of those living with PHIV and their HIV-exposed uninfected peers (HEU) had ever experienced IPV-victimization by a median of 21.8 years of age (range 18–28) with 65% reporting IPV in the past year [27,99]. In the US cohort, there was no difference in IPV prevalence by HIV status and IPV was associated with recent substance use, neighborhood stress, and poorer familial relationships [99]. While rates of IPV were lower in younger South African adolescents (lifetime IPV 37%, mean age 15 years, 76% PHIV), IPV and sexual abuse were strongly associated with poorer adherence to ART, an association previously reported in adults with NPHIV [100–102]. The relationship between victim and perpetrator is complex, with adolescent boys in South Africa who experienced violence themselves being more likely to use violence against their partners [103]. Additionally, adolescent boys with PHIV who experience IPV-victimization are more likely to report condomless sex with a detectable viral load, highlighting the need for IPV screening and interventions for both young men and women living with PHIV integrated within HIV care, both for the care and safety of the individual but also in the prevention of onward transmission [38]. Pregnancy is a particularly vulnerable time with IPV associated with increased rates miscarriage, still birth and impacts on mental health including suicidal ideation in the perinatal period for women living with NPHIV [104]. The impact of IPV is not limited to maternal health but impacts directly on vertical transmission with modeling, suggesting IPV is potentially responsible for one in eight infant HIV infections in sub-Saharan Africa, with rates highest in girls age 15–19 years [105].

## Pregnancy

Pregnant people with NPHIV have an increased risk of perinatal morbidity and mortality and while adverse perinatal outcomes are increased for those with viraemia, suppressive ART does not fully ameliorate the risk [106–108]. Despite prevention of vertical transmission of HIV, rates of low birth weight (LBW), small for gestational age (SGA), and preterm birth (PTB) remain elevated for infants born to those with NPHIV on suppressive ART prior to conception when compared to people not living with HIV in all settings, independent of ART class [108,109]. Emerging data for those with PHIV experiencing pregnancy suggest they are more likely to be younger, have detectable viraemia, immunosuppression, greater years of ART exposure and acquired HIV-1 drug resistance mutations when compared to pregnant people who have acquired HIV in later life [6,110–114]. In a US cohort, at delivery those with PHIV (median age 21 years) were more likely to have detectable viraemia (viral load >1000 c/ml 34% vs. 19%) and a CD4^+^ cell count less than 200 cells/ul (18% vs. 7%) compared to NPHIV (median age 30 years) [110]. In both US and UK cohorts, pregnant people with PHIV were a decade younger than those with NPHIV and adolescent pregnancy is commonly reported in PHIV cohorts across sub-Saharan Africa and Asia [6,110,113,114]. In South Africa, more than three quarters of pregnancies in those with PHIV occurred in adolescents of 19 years or less with almost half of adolescent pregnancies occurring 16 years of age or less, and comparable to those with PHIV delivering in Thailand and Vietnam where the median maternal age was 17.7 years [113,114]. In the general population, younger maternal age is an independent risk factor for adverse perinatal outcomes with adolescents (10–19 years) having a higher risk of preeclampsia and premature delivery of low-birth-weight infants when compared to young adults (20–24 years) with the risk further increased for adolescents living with HIV [115,116]. Adolescents living with HIV in Botswana were more likely to have preterm, SGA infants when compared to both aged matched adolescent peers not living with HIV and adults living with HIV [117]. Increased rates of premature SGA infants requiring enhanced neonatal care were also born to young people living with PHIV when compared to aged and ethnically matched peers not living with HIV in the UK [6]. While high-income settings have extremely small numbers of infants born to those living with PHIV, the real burden of adverse neonatal outcomes in this population will be seen in sub-Saharan Africa. Sub-Saharan Africa has the highest global neonatal mortality rates and a large systematic review in the region estimated that between 1990 and 2020, 1.9 million PTBs and 2.0 million SGA infants were attributable to HIV and ART [118]. The impact of adverse perinatal outcomes is not limited to childhood; SGA at birth is associated with an increased risk of cardiovascular disease, diabetes, obesity, and lower cognitive performance in adult life [119].

Despite the challenges of maternal viraemia and immunosuppression faced by some, reassuringly rates of vertical transmission in PHIV are low, ranging from 2.2% in South Africa, 1.9% in Asia, 1.6% in Spain, 1% in the USA, 0.6% in Brazil, and 0% in some European cohorts [6,110–114]. However, the impact of advanced maternal PHIV disease results in an increased rate of all-cause maternal mortality postpartum, the vast majority directly attributable to HIV and creates the next generation of orphaned as the result of living in a family with HIV. In the US, mortality postpartum was reported in 11% of PHIV, and after adjusting for age, the survival rates for NPHIV were 3.23 that of mothers with PHIV [110]. In Spain, 36% of those with PHIV dying (median age 25.8 years) following transition to adult care were already mothers [120]. ln Zimbabwe in a 6-year follow-up of a small cohort of mothers with PHIV (age 17–24 years), 16% had died and a further 16% were no longer engaged in care [121]. Retention in care postpartum is lower in adolescents when compared to both older mothers and to nonpregnant aged matched peers living with HIV and targeted interventions to improve retention during this vulnerable period are critically required [122,123]. In all settings, emerging data suggest, deaths following transition to adult care are predictable in earlier adolescence with unsuppressed viremia, immunosuppression and a prior CDC-C diagnosis on leaving pediatric care predictive of mortality in adult care [110,120,121,124,125].

For those who choose to delay parenthood into their 30 s and beyond, the long-term morbidity associated with lifelong exposure to HIV and ART has the potential to further impact pregnancy outcomes [5]. In the US by the age of 30 years, 19% of those living with PHIV have diabetes, 25% chronic kidney disease, and 22% hypertension [126]. Diabetes, hypertension, and chronic kidney disease increase both maternal and infant mortality and result in long-term morbidity associated with increased rates of prematurity and fetal growth restriction [127,128]. Mental health outcomes for this unique population also have the potential to impact pregnancy with condomless sex, substance use, poor adherence, and HIV viraemia associated with increasing psychiatric comorbidity [5,129,130]. Rates of depression, anxiety, and psychosis are generally reported as higher in those with PHIV when compared to the general population, and have the potential to impact adversely on perinatal mental health, ART adherence, and retention in care postpartum and are further complicated by HIV-stigma [5,131,132]. Young parents with PHIV cite the importance of the legacy of having children living free of HIV but frequently face clinical, educational, social, and psychological challenges when caring for their infants, and highlight the importance of support from family, peers, healthcare providers, and the wider society during this time if they and their infants are to thrive [133–135].

## HIV-exposed uninfected infants born to people with PHIV

Children who are HIV exposed but uninfected born to women with NPHIV have increased mortality, estimated at 1.8 times greater than infants not exposed to HIV in the first 12 months of life, morbidity, including infections requiring hospitalization, neurocognitive outcomes, growth, and undernutrition compared to children not exposed to HIV in the perinatal period [136–140]. The etiologies are complex, likely multifactorial and include the impacts of poor maternal health, in-utero ART exposure, prematurity, SGA, immune dysregulation and inflammation, reduced breast feeding, maternal mental health, food insecurity, and an adverse socioeconomic environment [136–141]. Longer term outcome data for the “3^rd^ generation” of HIV-exposed uninfected (HEU) infants born to women with PHIV are sparse, with early data suggesting potential deficits in speech and language acquisition and poorer growth out to 7 years associated with maternal viremia and SGA [142–144]. In a small US cohort, rates of hospitalization due to infectious causes were higher in HEU-PHIV when compared to HEU-NPHIV (aOR = 7.45, 95% CI: 1.58–35.04) [145]. Early data suggest differences in immune activation profiles in pregnancy for those living with PHIV compared to NPHIV but real understanding of both the potential drivers, and the magnitude of differences between children born to PHIV and NPHIV requires research data disaggregated by maternal route of HIV acquisition if interventions targeting the most vulnerable are to be developed [146,147].

## Data gaps and research priorities

**Fig. 1:**
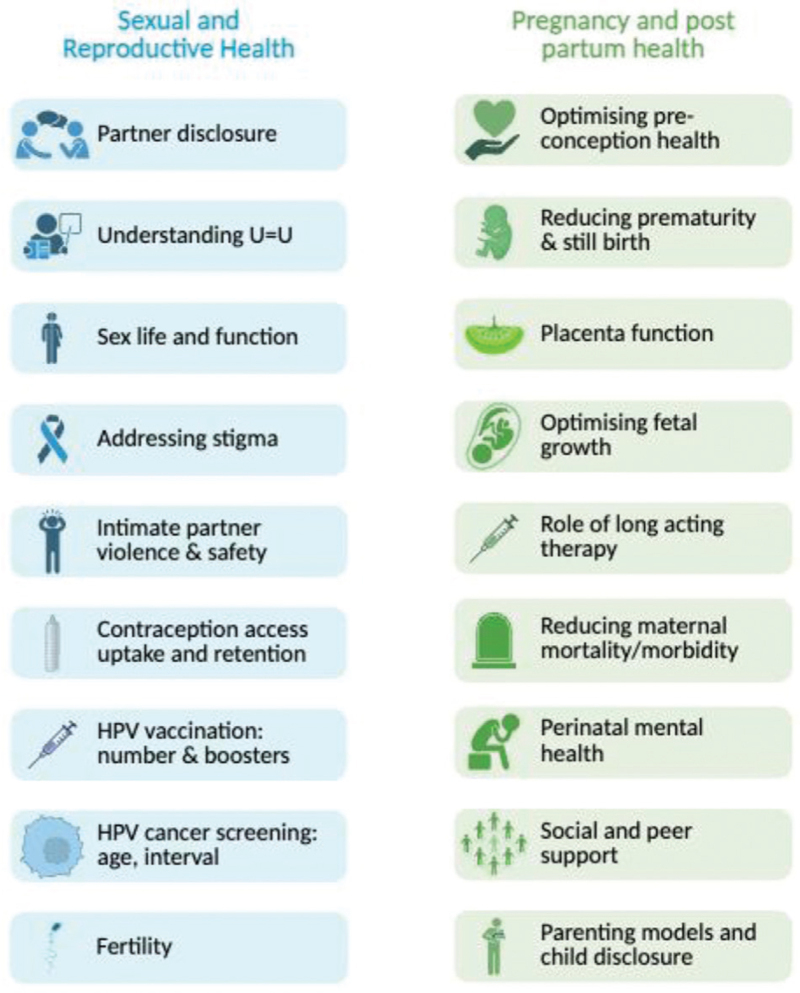
Data gaps and research priorities.

Some of the data gaps and resulting research priorities in SRH for the unique cohort who have lived their whole lives with HIV are highlighted in Fig. 1. While enormous gains have been made in the last 40 years, progress has stalled and young adults and pregnant people living with PHIV remain highly vulnerable, frequently excluded from research and without access to services that meet their needs. They are an extraordinary group of young people who show a huge amount of resilience in living well with HIV, and compassion toward their peers who struggle. They deserve equitable access to nonjudgmental SRH services, integrated within HIV and mental healthcare, tailored to their needs, voiced by them. Ultimately, ending the HIV epidemic will never be an attainable goal if young people with PHIV are left behind.

## Acknowledgements

### Conflicts of interest

There are no conflicts of interest.
